# Right ventricular electromechanical dyssynchrony in adults with repaired Tetralogy of Fallot

**DOI:** 10.3389/fped.2023.1085730

**Published:** 2023-02-23

**Authors:** Daniel Bowen, Martijn Kauling, Bernardo Loff Barreto, Jackie McGhie, Judith Cuypers, Tamas Szili-Torok, Jolien Roos-Hesselink, Annemien van den Bosch

**Affiliations:** Department of Cardiology, Erasmus Medical Center, Rotterdam, Netherlands

**Keywords:** tetralogy of fallot (TOF), RV dyssynchrony, electromechanical delay, right ventricular strain, 2D multi-plane echocardiography

## Abstract

**Background and purpose:**

Electromechanical dyssynchrony, manifested by right bundle branch block and regional wall mechanical dysfunction, contributes to inefficient RV function in repaired Tetralogy of Fallot (ToF). This study aims to evaluate the synchronicity of multiple RV walls using two-dimensional multi-plane echocardiography (2D-MPE) in order to augment current understanding of the mechanisms behind RV dyssynchrony.

**Methods:**

Sixty-nine adult ToF patients [aged 33 (23–45) years; 61% male] and twenty-five matched healthy controls underwent deformational analysis of the RV lateral, anterior, inferior and septal walls following 2D-MPE acquisitions. RV synchronicity was assessed by the intra-RV deformation delay between each basal RV wall and mid-septal segment in addition to mechanical dispersion calculated across four, six and eight segments (MD).

**Results:**

All RV wall-septum delays plus MD-4 and MD-6 indices were significantly greater in ToF patients compared to healthy controls (*p *< 0.001–0.03). In ToF patients, the lateral and anterior RV walls were last to reach peak deformation and anterior wall longitudinal strain was lower (*p* = 0.001). Post systolic shortening of at least one RV wall segment was identified in 19 (28%) ToF patients. Despite similar ECG characteristics, lateral and anterior wall-septum delays were significantly longer in patients with greater degrees of dyssynchrony (73 [37–108]ms vs. 37 [0–63]ms, *p* = 0.006; 91 [52-116]ms vs. 41 [1–69]ms, *p* = 0.013), although RV ejection fraction (RVEF) was not significantly lower. MD-4 and MD-8 indices displayed moderate negative associations with RVEF, strengthened by inclusion of lateral wall longitudinal strain (*r* = 0.64/0.65; *p* ≤0.01).

**Conclusion:**

RV dyssynchrony in ToF is characterised by electromechanical delays between the lateral, anterior and septal walls, with anterior wall dysfunction likely associated with surgical repair of the RV outflow tract. Prospectively, 2D-MPE may have an emerging role evaluating RV mechanical response to electrical resynchronisation therapy

## 1. Introduction

Right ventricular (RV) functional assessment in adults with repaired Tetralogy of Fallot (ToF) is challenging and complicated by conduction aberrations, regional wall dyskinesia following surgical repair and remodelling due to residual pulmonary valve regurgitation (1). RV electromechanical dyssynchrony may develop as a result of these factors and is thought to lead to progressive dysfunction and increased risk of ventricular arrhythmias (2, 3). QRS prolongation secondary to right bundle branch block (RBBB) causes early septal depolarization and delayed activation of the RV free wall (4, 5). Mechanically, an abrupt rightward septal movement in early systole, with concomitant stretch and subsequent post-systolic shortening of the basal RV free wall reduces effective shortening time (3). As a result, increased mechanical dispersion within the RV segments contributes to an inefficient RV contraction pattern and compromises diastolic filling (1, 6). In recent years, speckle tracking echocardiography (STE) has emerged as a modality to quantify RV dyssynchrony. From an apical four-chamber echocardiographic view, RV deformational timings are measureable and mechanical dispersion amongst the RV lateral wall and septal segments can be derived (3–8). However, this single plane two-dimensional approach overlooks regional RV wall abnormalities which are present in ToF patients. The anterior outflow tract region is frequently dyskinetic following initial repair and/or subsequent valvular intervention, whilst other free wall regions may be impacted by subsequent remodelling (9, 10). Using novel two-dimensional multi-plane echocardiographic imaging (2D-MPE), the anterior and inferior RV free wall regions can be evaluated in addition to the lateral wall. Acquisitions are made from one apical acoustic window using electronic plane rotation at suitable temporal resolutions for deformational analysis (11, 12). This study therefore aims to evaluate the synchronicity of multiple RV walls in order to augment current understanding of the mechanisms behind RV dyssynchrony.

## 2. Methods

### 2.1. Study population

ToF patients who had undergone detailed imaging of the RV with 2D-MPE during routine outpatient follow up at our ACHD centre were included in this study. In addition to two- and three-dimensional trans-thoracic echocardiogram (2D/3D-TTE), all individuals on the same day had a 12 lead electrocardiogram (ECG). Cardiovascular magnetic resonance (CMR), Holter and exercise test data were included if performed within ninety days of the TTE and ECG. Patients were excluded from analysis when RV free wall strain was only measurable in one wall. A control group of age and gender matched healthy individuals was used for comparison of RV deformational and synchronicity parameters, none of whom underwent CMR. The study was carried out according to the principles of the Declaration of Helsinki, was approved by the local medical ethics committee and written informed consent was obtained from all subjects.

### 2.2. Echocardiographic acquisition and conventional measurements

An extensive TTE protocol was carried out according to international guidelines (13) with additional focus on RV structure and function by acquiring 2D-MPE and 3D-TTE recordings. All TTEs were performed by sonographers specialised in congenital echocardiography. Studies were acquired using an EPIQ7 ultrasound system (Philips Medical Systems, Best, The Netherlands) equipped with an X5-1 matrix array transducer (composed of 3,040 elements with 1–5 MHz). Spatial and temporal resolution were optimised for 2D RV focused images in order to perform strain analysis offline. 3D recordings of the right heart were either multi-beat full volume acquisitions or made with single beat HeartModel software (Philips Medical Systems). Recordings were optimised to include the right ventricle at the highest possible volume rate with slight over gaining of the 2D image. Conventional 2D echocardiographic parameters for left and right heart size and function were collected in addition to the grading of any valvular lesions as either less than (<) or equal or greater than (≥) moderate in severity using parameters as documented in published guidelines (14, 15). RV basal, mid and longitudinal linear dimensions alongside fractional area change (FAC) were measured in the standard focused RV apical four chamber view. Tricuspid annular plane systolic excursion (TAPSE) and tissue Doppler imaging derived tricuspid annular peak systolic velocity (RV-S') were measured at the basal RV lateral wall.

### 2.3. Advanced right ventricular assessment by 2D multi-plane and 3D echocardiography

**Figure 1:**
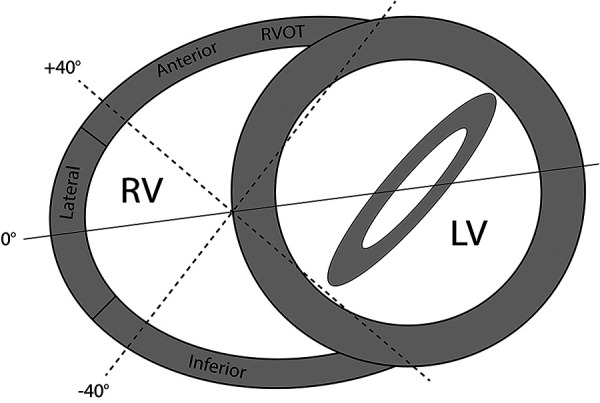
Multi-plane imaging of the right ventricle (RV) using 2D multi-plane echocardiography. Views obtained by electronic plane rotation around a single RV focused apical echocardiographic position. 0° rotation: lateral wall; +40°: anterior wall; −40°: inferior wall.

The evaluation of regional RV wall function by 2D multi-plane echocardiography has been well documented in our previous publications (12, 16). In short, from a fixed apical probe position, electronic plane rotation around the RV apex allows visualisation of different RV free wall regions. Each RV wall is confirmed by the presence of a certain left-sided landmark associated with an approximate degree of electronic rotation. Throughout imaging, the RV should be non-foreshortened with the RV apex and interventricular septum centred along or as near to the midline of the imaging sector as possible. For this study, three views were utilised visualising the lateral, anterior and inferior RV wall regions. The first view at 0˚ shows the lateral RV wall with the left sided landmark being the mitral valve. The second view at approximately +40˚ shows the anterior RV wall and the coronary sinus and thirdly at approximately −40˚ the inferior RV wall and the aortic valve (Figure 1, Supplementary Videos S1–S3). The RV datasets were digitally exported to a vendor-neutral server (TomTec Imaging Systems, Unterschleissheim, Germany) and data analysis was performed offline by one experienced observer (DB). To assess peak systolic RV longitudinal strain, an RV algorithm wall motion tracking software was used (2D CPA, Image-Arena version 4.6; TomTec Imaging Systems). RV systole was determined as the time interval from electrocardiographic QRS onset to minimum RV cavity size, which was used as a surrogate for pulmonary valve closure. The endocardial borders of the RV free wall and septum were manually traced at end systole and adjusted accordingly in end diastole if required. This was performed in each of the other multi-plane views previously described. A single segment RV longitudinal strain (RV-LS) value for each wall was derived from the average of the basal, mid and apical segments. A measurement was considered feasible if all portions of the RV wall tracked acceptably throughout the cardiac cycle. If tracking was deemed inaccurate, the wall was excluded from analysis. The 3D datasets were digitally exported to the same TomTec server and analysed by DB using specialised RV analysis software (TomTec 4D-RV function 2.0). After placing set landmarks, RV volumes and ejection fraction (RVEF) were automatically calculated, with manual adjustment performed where necessary. In cases of inadequate tracking, the dataset was deemed unfeasible to measure and excluded from analysis.

### 2.4. Measures of electromechanical dyssynchrony

**Figure 2:**
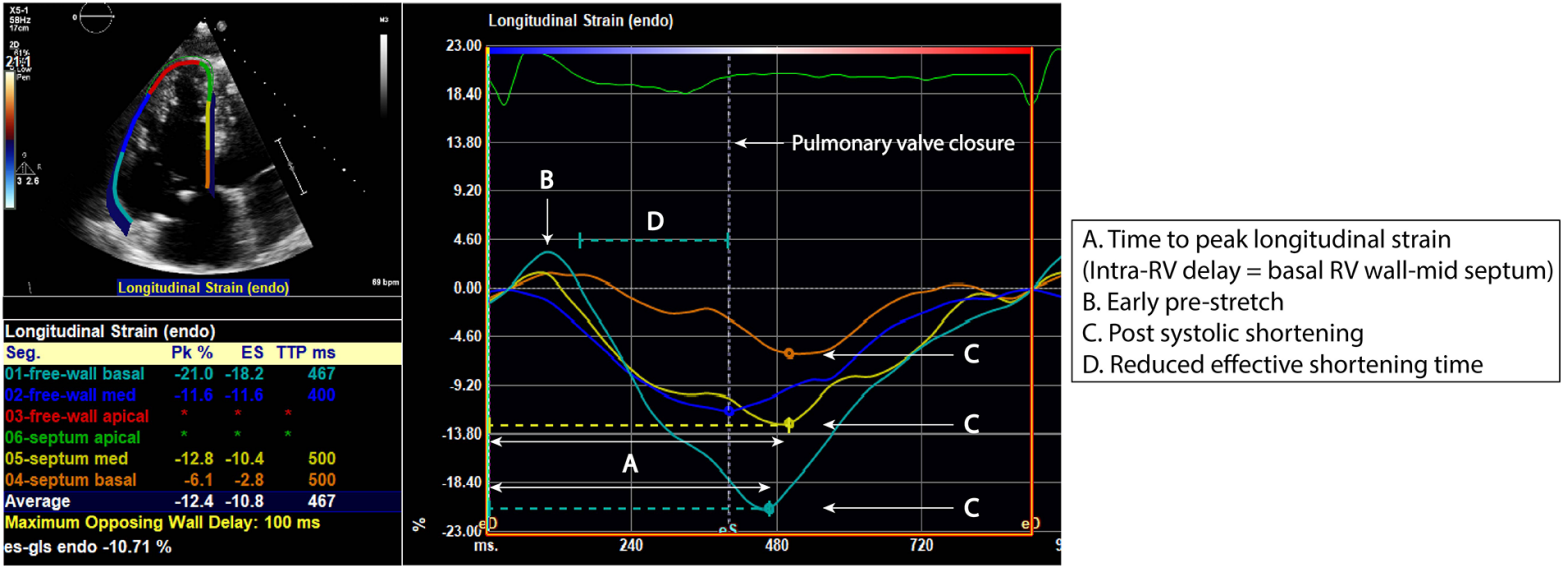
RV segmental strain curves in a repaired ToF patient with evidence of right ventricular (RV) dyssynchrony. Time to peak longitudinal strain (**A**) of the RV lateral wall and septum is measured at the basal and mid segments. There is presence of basal lateral wall pre-stretch (**B**) and post systolic shortening of the basal lateral wall and basal to mid septum (**C**). These characteristics result in a reduced effective shortening time (**D**).

Parameters used to measure RV dyssynchrony are detailed in Table 1, Figure 2. Speckle tracking (STE) analysis of the basal and mid segments of the RV walls and septum were used with the apical segments excluded owing to high variability reported by previous studies (7). The time to peak longitudinal strain value (TTP-LS) of each segment was corrected for heart rate ([timing/R-R interval]×1000). RBBB-induced intra-RV depolarization delay results in early activation of the septum and late activation of the RV free wall, with the basal segment last to contract. In concurrence with the study by Hui et al. (6), intra-RV deformation delay was quantified as the difference in TTP-LS between the basal RV wall and mid-septum (RV wall-septum delay). Mechanical dispersion was calculated as the standard deviation of average TTP-LS values across the four basal to mid segments of the septum and RV lateral wall (MD-4). Furthermore, a six and eight segment “global” mechanical dispersion value was calculated when TTP-LS of other RV wall basal-mid segments were measureable in an individual (MD-6: inclusive anterior or inferior wall; MD-8: inclusive anterior and inferior walls). Significant post systolic shortening (PSS) of the basal RV wall segment was identified if peak strain occurred two or more frames following the end systolic marker. Significant pre-stretch (PS) was determined visually whereby the curve of the basal RV segment demonstrated sustained positive strain in early systole. The presence of a greater degree of RV dyssynchrony in ToF patients was determined using the upper 95% limit of normal (mean ± 2SD) of MD-4 values from the control group.

**Table 1:** Definitions of RV dyssynchrony parameters.

Variable	Description
Time to peak longitudinal strain (TTP-LS)	Time from QRS onset to peak myocardial longitudinal shortening.
Intra-RV deformation delay (RV wall-septum delay)	Difference in TTP-LS between the basal RV wall and mid-septal segments.
Mechanical dispersion-4 (MD-4)	Standard deviation of average TTP-LS values across four basal-mid RV segments (septum and lateral wall).
Mechanical dispersion-6 (MD-6)	Standard deviation of average TTP-LS values across six basal-mid RV segments (inclusive anterior or inferior wall).
Mechanical dispersion-8 (MD-8)	Standard deviation of average TTP-LS values across eight basal-mid RV segments (inclusive anterior and inferior walls).
Pre-stretch (PS)	Myocardial lengthening (positive strain) of the basal RV wall segment in early systole.
Post systolic shortening (PSS)	Continuation of myocardial shortening after pulmonary valve closure, prominently seen in the basal RV wall segment.

### 2.5. Cardiovascular magnetic resonance

CMR images were acquired using a Sigma 1.5-T whole-body scanner (GE Medical Systems, Milwaukee, WI) with dedicated phased-array cardiac surface coils. Details of the MR sequence used have been reported previously (17). Analysis of the CMR sequence was performed using a commercially available Advanced Windows workstation (GE Medical Systems) equipped with Q-mass (version 5.2, Medis Medical Imaging Systems, Leiden, the Netherlands). The ventricular volumetric dataset was quantitatively analysed by manually outlining RV endocardial borders in end systole and end diastole, excluding large trabeculae and the papillary muscles from the blood volume. RV end-diastolic and end-systolic volumes (indexed for body surface area), plus RV ejection fraction (RVEF) were calculated.

### 2.6. Clinical parameters

Clinically relevant arrhythmias were defined as the presence of >100 isolated ventricular ectopic beats or >20 couplets/non-sustained runs documented over a 24 h period (18). Exercise tolerance was presented using results from exercise-ECG or cardiopulmonary exercise testing (CPET). The percentage of predicted physical work capacity and peak oxygen consumption were collected. Exercise intolerance was defined as <70% of one of the respective predictive peak values.

#### 2.6.1. Statistical analysis

The distribution of data was assessed using histograms and the Shapiro–Wilk test. Depending on the data distribution, continuous data is presented as mean ± standard deviation (SD) or median [inter-quartile range], whilst categorical data is presented as frequencies and percentages. For comparison of normally distributed continuous variables the independent samples T-test was used and in case of skewed distribution, the Mann–Whitney U-test was applied. For comparison of frequencies the X² test was used. The paired T-test was used for comparison of within-subject continuous parameters which were normally distributed, whilst the Wilcoxon Signed Rank test was used in case of skewed distribution. The Pearson correlation coefficient (r) was used to correlate measures of RV mechanical dispersion with CMR-derived RVEF. Multivariable linear regression analysis was undertaken with selected parameters (QRS duration; incidence of pulmonary valve re-intervention; trans-annular patch at initial repair; degree of pulmonary regurgitation; RV lateral wall strain). If parameters were suspected to be collinear, the multivariable analysis only included the parameter with the highest univariable correlation. Intra-observer agreement was assessed by repeated analysis in a random sample of twenty ToF study subjects performed at least six months after the initial analysis and blinded to the initial results. Assessment of inter-observer agreement was performed by a second experienced observer in the same sample (BB). The agreement between two measurements was determined as the mean of the differences +1.96 SD. Additionally, the intra-class correlation coefficient (ICC) was provided. All statistical analyses were performed using the Statistical Package for Social Sciences version 25 (SPSS, Inc., Armonk, NY, USA). The statistical tests were two-sided and a *p*-value < 0.05 was considered statistically significant.

## 3. Results

### 3.1. Demographics

**Figure 3:**
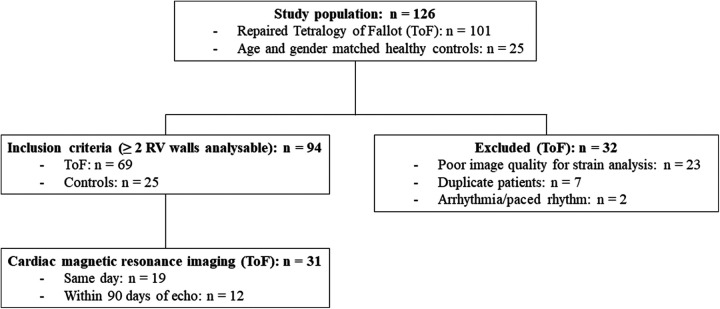
Study inclusion.

One hundred and one ToF patients who had undergone detailed imaging of the RV with 2D-MPE in our ACHD centre were evaluated for study inclusion. Sixty-nine patients [age = 33 (23–45) years; 61% male] were ultimately included in the study whereby RV free wall strain was measurable in two or more RV walls, in addition to the RV septum. Thirty-two patients were excluded, predominantly due to poor image quality for strain analysis (Figure 3). Thirty-one (45%) patients had undergone CMR within ninety days of TTE, of which nineteen (61%) were same day examinations. Holter data was available in 25 (36%) patients, exercise-ECG in 34 (49%) and CPET in 19 (28%). Thirty-six (52%) patients had a transannular patch at initial repair, whilst thirty (43%) patients had undergone at least one subsequent pulmonary valve re-intervention. Twenty-five healthy individuals [age = 34 (25–46) years; 52% male] were included in the control group.

### 3.2. Baseline characteristics

Demographic, ECG, CMR and TTE data are detailed in Table 2. There were no significant differences in age, gender or body mass index between patients and control subjects. Forty-eight (71%) ToF patients had RBBB on ECG, eleven (16%) had RBBB plus left anterior fascicular block, whilst the remaining nine (13%) patients had no clear bundle branch block pattern. The number of ToF patients with ≥ moderate right sided valve disease was as follows: pulmonary valve insufficiency – 35 (51%); pulmonary valve stenosis – 8 (12%); tricuspid regurgitation – 4 (6%). Left ventricular systolic function was widely preserved [*n* = 67 (97%)]. Of the ToF patients who underwent CMR, RVEF was 46 ± 9%.

**Table 2:** Baseline characteristics and echocardiography.

	Repaired ToF (*n* = 69)	Controls (*n* = 25)	*p*-value
**Demographics**			
Age	33 [23, 45]	34 [25, 46]	0.79
Male gender, *n* (%)	42 (61)	13 (52)	0.44
Body mass index, kg/m²	23.0 ± 3.0	23.7 ± 2.9	0.31
Systolic blood pressure, mmHg	123 ± 15	129 ± 14	0.11
Diastolic blood pressure, mmHg	77 ± 12	79 ± 11	0.53
**Electrocardiogram**^**a**^			
Right bundle branch block (RBBB), *n* (%)	48 (71)	-	
RBBB and left anterior fascicular block, *n* (%)	11 (16)	-	
No bundle branch block, *n* (%)	9 (13)	25 (100)	**<0.001**
R-axis, degrees	78 [16, 96]	70 [51, 81]	0.27
QRS duration, ms	139 ± 29	98 ± 14	**<0.001**
**Cardiothoracic intervention**			
Age at initial surgical correction, years	1.2 [0.6, 4.3]	-	
Transannular patch at initial repair, *n* (%)	36 (52)	-	
Pulmonary valve re-intervention, *n* (%)	30 (43)	-	
Age at pulmonary valve re-intervention, years	26 ± 12	-	
**Cardiovascular magnetic resonance, *n* = 31**			
RV end diastolic volume index, ml/m²	121 [98, 156]	-	
RV end systolic volume index, ml/m²	61 [50, 77]	-	
RV ejection fraction, %	46 ± 9	-	
**Transthoracic echocardiography**			
Right ventricular basal dimension, mm	47 ± 9	39 ± 6	**<0.001**
Right ventricular longitudinal dimension, mm	91 ± 10	86 ± 9	**0.015**
Right ventricular outflow tract 1 dimension, mm	39 [35, 44]	31 [29, 32]	**<0.001**
Right atrial area, cm²	21 [18, 25]	16 [15, 19]	**<0.001**
TAPSE, mm	17 ± 4	26 ± 5	**<0.001**
RV S’, cm/s	10 ± 3	13 ± 2	**<0.001**
Fractional area change, %	34 ± 8	43 ± 5	**<0.001**
3D right ventricular end diastolic volume index, ml/m²^b^	96 [75, 123]	56 [50, 71]	**<0.001**
3D right ventricular end systolic volume index, ml/m²^b^	51 [39, 70]	23 [20, 30]	**<0.001**
3D right ventricular ejection fraction, %^b^	45 [40, 49]	58 [58, 62]	**<0.001**
≥ Moderate pulmonary regurgitation, *n* (%)	35 (51)	-	
≥ Moderate pulmonary stenosis, *n* (%)	8 (12)	-	
≥ Moderate tricuspid regurgitation, *n* (%)	4 (6)	-	
Tricuspid regurgitation maximum velocity, m/s	2.7 [2.5, 3.1]	2.1 [1.8, 2.4]	**<0.001**
Left ventricular systolic function, *n* (%):			
Preserved	67 (97)	25 (100.0)	0.39
Significantly impaired (LVEF ≤45%)	2 (3)	-	** **
**Left ventricular diastolic function, *n* (%)**			
Normal	42 (61)	21 (84)	**0.035**
Impaired relaxation	6 (9)	4 (16)	0.31
Pseudo-normal	8 (12)	-	** **
Restrictive filling	2 (3)	-	** **
Unclear	11 (16)	-	** **
Medial E/e’ ratio	10 [8, 13]	7 [6, 8]	**<0.001**
Left atrial diameter, mm	40 [38, 44]	35 [32, 38]	**<0.001**

^a^ One ToF patient without available ECG data.

^b^ 3D echo data measureable: ToF - 47 (68.1%), controls - 21 (84%).

### 3.3. RV multi-wall deformation and synchronicity

**Figure 4:**
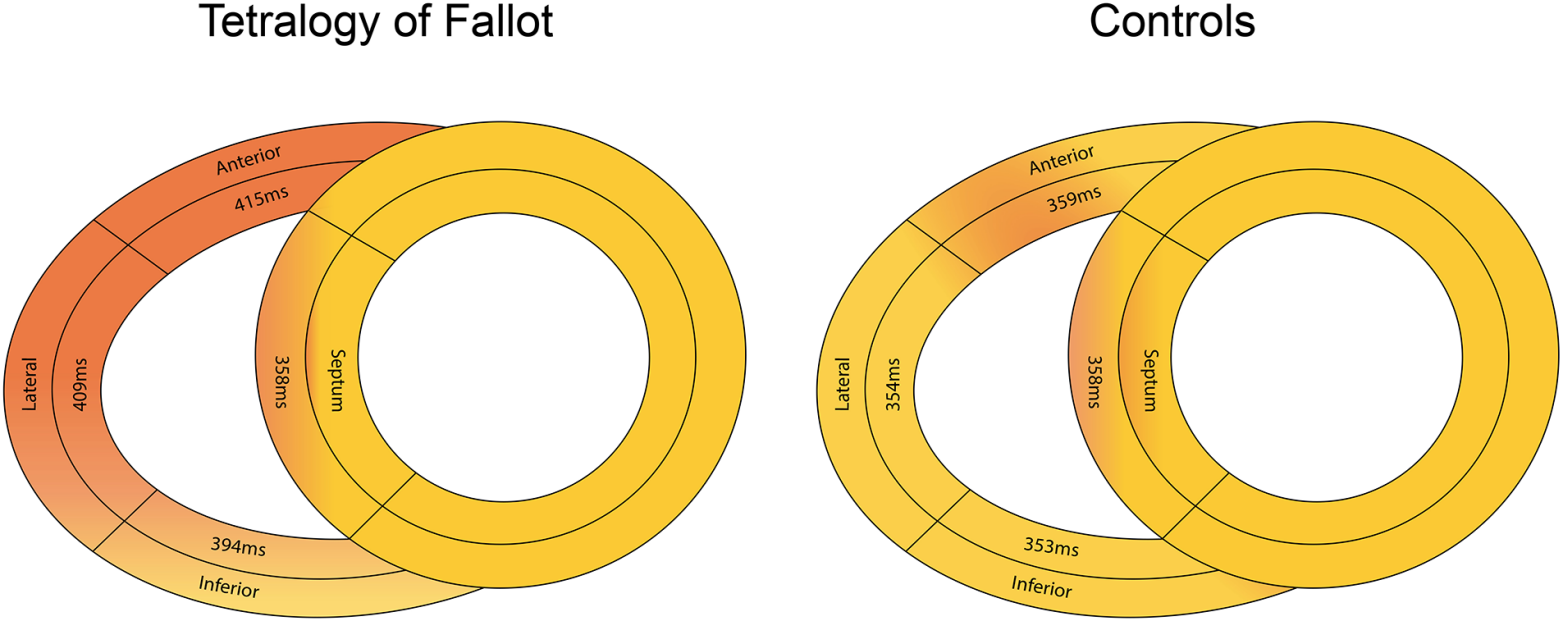
Differences in median time to peak longitudinal strain values across the basal right ventricular free wall and mid-septum in ToF patients (left panel) and healthy controls (right panel).

Table 3 demonstrates all multi-wall longitudinal strain and synchronicity parameters in ToF patients and healthy controls. Whilst lateral wall LS was performed in all ToF patients, measurement feasibility was lower for the anterior [52 patients (75%)] and inferior walls [65 (94%)]. All RV walls were measureable in 48 (70%) patients and all healthy controls. LS values of all RV walls and septum were significantly lower in ToF patients compared to controls (*p* < 0.001). In ToF patients, LS was lower in the anterior wall (−18 ± 4%) compared to the lateral [−20 ± 4% (*p* = 0.001)] and inferior walls [−20 ± 4% (*p* = 0.008)]. Figure 4 demonstrates TTP-LS values across the basal RV walls and mid septum. In controls, TTP-LS occurred almost simultaneously across these segments, whilst in ToF patients regional differences were evident. In ToF, TTP-LS of all RV walls was significantly longer than that of the septum (*p* < 0.001). Furthermore, TTP-LS was longer in the lateral (409 ± 68 ms) and anterior (415 ± 80 ms) walls compared to the inferior wall [394 ± 68 ms (anterior vs. inferior wall, *p* = 0.04)]. This resulted in long intra-RV delays between the basal wall and mid-septal segments (lateral wall: 60 [19, 86] ms; anterior wall: 64 [19, 99] ms; inferior wall: 40 [−1, 82] ms). MD-4 and MD-6 values were significantly higher in ToF patients compared to healthy controls (MD-4: *p* < 0.001; MD-6: *p* = 0.03), whilst MD-8 values were not significantly different. PSS of at least one RV wall segment was identified in nineteen (28%) ToF patients and one control subject (4%, *p* = 0.01). PS was less prevalent, identified in nine (13%) ToF patients and one control subject (4%). Both PS and PSS were present in five (7%) ToF patients.

**Table 3:** RV deformation and synchronicity parameters in ToF patients and controls.

	Repaired ToF (*n* = 69)	Controls (*n* = 25)	*p*-value
**Multi-plane echocardiographic assessment**			
RV septum longitudinal strain (LS), %	−14 ± 4	−20 ± 3	**<0.001**
RV lateral wall LS, %	−20 ± 4	−28 ± 3	**<0.001**
RV anterior wall LS, %	−18 ± 4 ^(52)^	−28 ± 4	**<0.001**
RV inferior wall LS, %	−20 ± 4 ^(65)^	−27 ± 5	**<0.001**
**RV synchronicity assessment**			
RV mid septum time to peak LS (TTP-LS), ms	358 ± 63	358 ± 63	0.95
RV basal lateral wall TTP-LS, ms	409 ± 68	354 ± 55	**<0.001**
RV basal anterior wall TTP-LS, ms	415 ± 80 ^(52)^	359 ± 57	**<0.001**
RV basal inferior wall TTP-LS, ms	394 ± 68 ^(65)^	353 ± 61	**0.008**
Lateral wall-septum delay, ms	60 [19, 86]	0 [−28, 24]	**<0.001**
Anterior wall-septum delay, ms	64 [19, 99] ^(52)^	0 [−32, 44]	**<0.001**
Inferior wall-septum delay, ms	40 [−1, 82] ^(65)^	−10 [−37, 11]	**0.002**
Mechanical dispersion (4 segments), ms	40 [24, 46]	17 [12, 24]	**<0.001**
Mechanical dispersion (6 segments), ms	36 [26, 49]	29 [18, 37]	**0.034**
Mechanical dispersion (8 segments), ms	35 [27, 50] ^(48)^	30 [20, 41]	0.07
Evidence of pre-stretch in ≥1 RV wall, *n* (%)	9 (13)	1 (4)	0.21
Evidence of post systolic shortening in ≥1 RV wall, *n* (%)	19 (28)	1 (4)	**0.014**
Evidence of PS and PSS in ≥1 RV wall, *n* (%)	5 (7)	0 (0)	0.17

Time to peak longitudinal strain (TTP-LS) values corrected for heart rate (TTP/R-R interval × 1000). Feasible measurements are noted in subscript (*n*), other measurements were 100% feasible.

### 3.4. RV dyssynchrony in ToF

**Figure 5:**
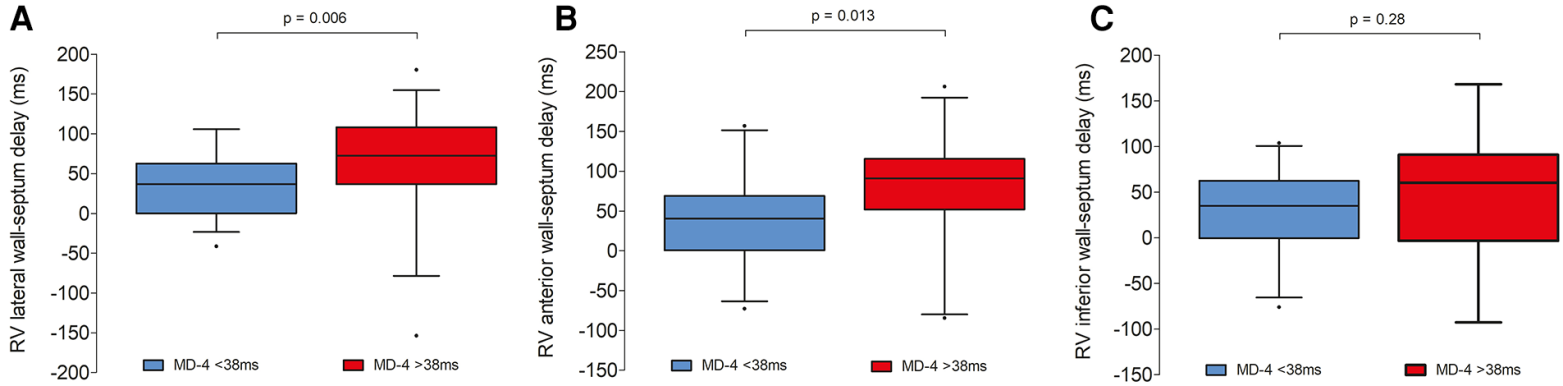
Box and whisker plots demonstrating intra-RV delays between each basal RV wall and mid septal segment in ToF patients with and without greater degree of RV dyssynchrony [mechanical dispersion (MD-4) value >38 ms]. Left panel (**A**): RV lateral wall; middle panel (**B**): RV anterior wall; right panel (**C**): RV inferior wall.

A greater degree of RV dyssynchrony in ToF patients was defined as an MD-4 value above 38 ms. According to this criterion, greater RV dyssynchrony was present in 38 (55%) patients (Table 4). There were no significant differences in QRS duration or bundle branch block type in patients with or without greater dyssynchrony, nor for incidence of pulmonary valve re-intervention or degree of pulmonary regurgitation. Incidence of clinically relevant arrhythmias was similar between groups where Holter data was available (43% of patients with MD-4 > 38 ms vs. 55% with MD-4 < 38 ms, *p* = 0.56). Exercise intolerance from available exercise–ECG or CPET was evident in one patient with greater RV dyssynchrony and in two patients with less RV dyssynchrony (*p* = 0.77). There was no relationship between percentage of predicted peak oxygen consumption and MD-4 value (*r* = −0.14, *p* = 0.57). Lateral and anterior wall-septum delays were significantly longer in ToF patients with greater RV dyssynchrony (73 [37–108] ms vs. 37 [0–63] ms, *p* = 0.006; 91 [52–116] ms vs. 41 [1–69] ms, *p* = 0.013 – Figure 5). RV septum LS was also significantly lower (−13 ± 3% vs. −15 ± 4%, *p* = 0.034), although there were no significant differences in RV wall LS. Furthermore, there was a trend towards a higher incidence of PSS in patients with greater RV dyssynchrony (14 [37%] vs. 5 [16%], *p* = 0.06). These findings did not however translate to a significant difference between groups in RVEF as measured by either 3D-TTE (44 ± 6% vs. 47 ± 6%, *p* = 0.25) or CMR (46 ± 8% vs. 47 ± 9%, *p* = 0.54). In seventeen patients who underwent CMR with gadolinium, late gadolinium enhancement (LGE) was predominantly seen at the inferior RV insertion point. Four patients with an MD-4 value >38 ms also had extensive LGE in the RVOT, compared to none with MD-4 < 38 ms (*p* = 0.18).

**Table 4:** RV functional and synchronicity parameters in ToF patients with and without greater degree of RV dyssynchrony.

	MD-4 > 38 ms (*n* = 38)	MD-4 < 38 ms (*n* = 31)^a^	*p*-value
**Electrocardiogram and pulmonary valve**			
* *QRS duration, ms	136 ± 29	143 ± 29	0.32
* *Right bundle branch block (RBBB), *n* (%)	29 (76)	19 (63)	0.46
* *RBBB and left anterior fascicular block, *n* (%)	5 (13)	6 (20)	0.45
* *Trans-annular patch at initial repair, *n* (%)	20 (53)	16 (52)	0.93
Pulmonary valve re-intervention, *n* (%)	17 (45)	13 (42)	0.82
* *≥Moderate pulmonary regurgitation, *n* (%)	19 (50)	16 (52)	0.89
**Right ventricular function**			
RV ejection fraction (CMR), %	46 ± 8 ^(17)^	47 ± 9 ^(14)^	0.54
RV ejection fraction (3D-TTE), %	44 ± 6 ^(27)^	47 ± 6 ^(20)^	0.25
RV septum LS, %	−13 ± 3	−15 ± 4	**0.034**
RV lateral wall LS, %	−20 ± 4	−20 ± 5	0.83
RV anterior wall LS, %	−18 ± 4 ^(27)^	−18 ± 4^(25)^	0.95
RV inferior wall LS, %	−19 ± 4 ^(35)^	−20 ± 3^(30)^	0.75
**RV synchronicity assessment**			
Lateral wall-septum delay, ms	73 [37, 108]	37 [0, 63]	**0.006**
Anterior wall-septum delay, ms	91 [52, 116] ^(27)^	41 [1, 69]^(25)^	**0.013**
Inferior wall-septum delay, ms	60 [−3, 91] ^(35)^	35 [0, 62]^(30)^	0.28
Evidence of pre-stretch in ≥1 RV wall, *n* (%)	7 (18)	2 (6)	0.14
Evidence of post systolic shortening in ≥1 RV wall, *n* (%)	14 (37)	5 (16)	0.06

Mechanical dispersion (MD-4) value >38 ms indicative of greater degree of RV dyssynchrony.

^a^ One patient in MD-4 <38 ms group without available ECG data. Feasible measurements are noted in subscript (*n*). Other measurements were 100% feasible.

### 3.5. Association between RV dyssynchrony and RVEF

MD-4 and MD-8 values displayed moderate negative associations with CMR-derived RVEF (MD-4: *r* = −0.38, *p* = 0.017; MD-8: *r* = −0.51, *p* = 0.007). Associations were strengthened in a multiple regression model, predominantly by the inclusion of lateral wall longitudinal strain (MD-4: *r* = −0.64, *p* = <0.001; MD-8: *r* = −0.65, *p* = 0.006). Further inclusion of pulmonary regurgitation severity and incidence of pulmonary valve re-intervention only marginally increased the r value (MD-4: *r* = −0.67, *p* = 0.003; MD-8: *r* = −0.68, *p* = 0.026). Weaker associations were identified between 3D-TTE-derived RVEF and mechanical dispersion values (MD-4: *r* = −0.18, *p* = 0.22; MD-8: *r* = −0.30, *p* = 0.09). No associations were found between RV wall to septum delays and CMR-RVEF (*r* values <0.1). Furthermore, very weak associations were found between indices of RV dyssynchrony and QRS duration or with RV wall LS (all *r* values <0.2).

### 3.6. Intra- and inter-observer variability

All intra- and inter-observer agreements are presented in Table 5. Intra-observer agreement was excellent for all LS and timing parameters [ICC ≥ 0.7 except for RV inferior wall-septum delay (0.64)]. Inter-observer agreement for LS measurements was overall lower, although timing parameters were comparable and in the case of MD indices superior to intra-observer agreement (ICC ≥ 0.88).

**Table 5:** Intra- and inter-observer agreements of multi-RV wall strain and electromechanical dyssynchrony parameters.

	Intra-observer bias	95% limits of agreement	ICC	Inter-observer bias	95% limits of agreement	ICC
**Longitudinal strain (%)**						
Inter-ventricular septum	1.8	1.1	0.81*	2.3	1.9	0.79*
RV lateral wall	2.1	2.3	0.79*	3.2	2.4	0.84*
RV anterior wall	1.9	1.6	0.84*	4.5	2.6	0.62
RV inferior wall	2.3	2.2	0.79*	5.1	3.8	0.60
**RV dyssynchrony parameters (ms)**						
Lateral wall-septum delay, ms	25.3	24.6	0.76*	21.8	22.5	0.79*
Anterior wall-septum delay, ms	16.3	20.8	0.88*	22.4	23.8	0.84*
Inferior wall-septum delay, ms	25.3	30.8	0.64*	37.6	32.8	0.60
Mechanical dispersion (4 segments)	9.8	12.4	0.78*	7.2	5.7	0.91*
Mechanical dispersion (6 segments)	7.7	9.4	0.78*	7.4	5.5	0.88*
Mechanical dispersion (8 segments)	6.4	6.9	0.80*	6.3	4.1	0.88*

Performed on 20 ToF patients. ICC = Intraclass correlation coefficient. *p < 0.001.

## 5. Discussion

This is the first study evaluating RV dyssynchrony across multiple RV walls using speckle tracking echocardiography. 2D-MPE imaging provides a more global approach to RV wall deformational assessment and is particularly pertinent in ToF owing to the presence of regional wall abnormalities (16). We found differences in TTP-LS across all RV segments in ToF, leading to a dyssynchronous and inefficient contraction sequence in contrast to relatively simultaneous activation in healthy individuals. The lateral and anterior RV walls are last to reach peak deformation, resulting in longer electromechanical delays with the septum and increased intra-RV mechanical dispersion. Combined with reduced LS, there is impairment of the entire anterior wall, most likely associated with previous surgical repair of the RV outflow tract and subsequent RV remodelling. In a smaller subset of ToF patients who underwent CMR, mechanical dispersion indices displayed moderate negative associations with RVEF, although strengthened when the lateral wall LS was taken into consideration. A greater degree of RV dyssynchrony was therefore not consistently reflective of dysfunction and other TTE functional parameters should be supportive.

### 5.1. RV electromechanical function in ToF

Regional differences in TTP-LS and high intra-RV mechanical dispersion reflect the complex electromechanical factors present in ToF. TTP-LS occurred earliest in the RV septum and following delayed activation of the RV free wall, the basal inferior wall reached peak deformation before the lateral and anterior walls. Whilst RBBB is ubiquitous, QRS prolongation was not associated with indices of RV dyssynchrony. In cases of proximal block, RV depolarisation occurs *via* the anterior and posterior fascicles of the left bundle, lengthening QRS duration and delaying RV free wall activation (19). However, in an electrophysiological (EP) study of ToF patients, Verzaal et al. (20) demonstrated that functional blocks often occurred in the repaired RVOT region, whilst conduction properties of the working myocardium were fairly well preserved. Proximity to the dyskinetic RVOT is therefore a likely cause of the electromechanical abnormalities of the RV anterior wall. Post-operative RV remodelling also impacts intra-myocardial conduction and factors such as by scar size, patch material, degree of myocardial fibrosis and residual pulmonary valve regurgitation may have varying effects on electromechanical function across the RV segments (21–23). Seventeen CMR studies were performed with gadolinium which broadly demonstrated LGE at the inferior RV insertion point. There was however a higher incidence of RVOT LGE in patients with longer anterior wall to septum mechanical delays. This may indicate higher scar burden in patients with greater dyssynchrony, although needs to be demonstrated in a larger study.

A four-segment mechanical dispersion measurement (MD-4), reflecting differences in TTP-LS between basal to mid segments of the septum and lateral wall, compared favourably with that of the study by Yim et al. (3). It is however debatable whether extra RV wall segments are warranted for a more accurate calculation. Mechanical dispersion derived including the anterior and inferior wall segments (MD-6/8), remained stable in ToF patients but considerably increased in healthy individuals, suggestive of high variability. MD-4 seems suitable for use in daily practice given that lateral wall deformational timings are comparable to those of the anterior wall. The additional RV segments may however have more relevance in pre/post resynchronisation studies.

Segmental post systolic shortening after pulmonary valve closure was present in one third of ToF patients and was more prevalent in those with greater RV dyssynchrony. Pre-stretch was however less prevalent in our study population than has been described in RV dyssynchrony studies performed in children (3, 4, 6). This may be a consequence of lower imaging resolution in adult patients leading to subtleties in deformation being missed. Conversely, this could be due to selection bias in our sampled population, with fewer cases of significant RV dysfunction.

### 5.2. Clinical impact and future directions

As the population of ToF patients continues to grow and age worldwide, incidence of right ventricular failure is increasing and treatment options are limited. In cases of significant dysfunction associated with RV dyssynchrony, there is evidence that some patients may benefit from cardiac resynchronisation therapy (19). In EP studies, the RVOT has been identified as the latest region to be activated in a majority of ToF patients and RV apical pacing may therefore not be sufficient to rectify electrical delays (24–26). Resynchronisation of septal and RV free wall contraction by pacing from the area of latest RV activation can lead to an increase in contraction efficiency, improvement in diastolic filling and subsequent reverse remodelling (26–29). In this setting, 2D-MPE has a role in identifying regions of late mechanical activation and evaluating global RV response to electrical resynchronisation.

### 5.3. Study limitations

Limitations of this study include a relatively small sample size, particularly for comparison of RV dyssynchrony parameters with CMR-derived function. Furthermore, 14/31 (45%) patients underwent CMR without gadolinium, which limited the evaluation of corresponding scar burden in the RV myocardium. These findings should be interpreted on this basis and thus further robust research is required. RV deformation was only measured in the longitudinal direction and thus components of radial and circumferential shortening were not considered. Furthermore, LV deformation was not quantified therefore the effect of ventricular interdependence on RV mechanics (30, 31) was overlooked. Due to its retro-sternal position, it remains difficult to quantify subvalvular RVOT function with STE, therefore this region could not be evaluated. Multi-RV wall strain measurements were not made simultaneously as this is not possible with 2D-MPE imaging, however all timing indices were corrected for the heart rate on each acquisition. Technological advances may make 3D-STE of the RV more attractive in the coming years, enabling multi-RV wall deformational evaluation to be performed simultaneously and faster (32).

## 6. Conclusion

A high proportion of ToF patients will develop RV dysfunction following initial or subsequent reparative surgery and treatment options are limited. 2D-MPE imaging enables a more global quantification of RV deformation, providing insights into regional electromechanical function and mechanisms of dyssynchrony. We found that RV dyssynchrony is characterised by electromechanical delays between the lateral, anterior and septal walls, resulting in increased intra-RV mechanical dispersion. Prospectively, 2D-MPE imaging may have an emerging role evaluating RV mechanical response to electrical resynchronisation therapy.

## Data Availability

The original contributions presented in the study are included in the article/**Supplementary Material**, further inquiries can be directed to the corresponding author/s.

## Supplementary material

**SUPPLEMENTARY VIDEO S1**

Echocardiographic video loop of the mitral valve view demonstrating the right ventricular lateral wall (0º electronic rotation).

**SUPPLEMENTARY VIDEO S2**

Echocardiographic video loop of the coronary sinus view demonstrating the right ventricular anterior wall (approximately +40º electronic rotation).

**SUPPLEMENTARY VIDEO S3**

Echocardiographic video loop of the aortic valve view demonstrating the right ventricular inferior wall (approximately -40º electronic rotation).
